# Interferon-γ blocks signalling through PDGFRβ in human brain pericytes

**DOI:** 10.1186/s12974-016-0722-4

**Published:** 2016-09-21

**Authors:** Deidre Jansson, Emma L. Scotter, Justin Rustenhoven, Natacha Coppieters, Leon C. D. Smyth, Robyn L. Oldfield, Peter S. Bergin, Edward W. Mee, E. Scott Graham, Richard L. M. Faull, Mike Dragunow

**Affiliations:** 1 Department of Pharmacology and Clinical Pharmacology, The University of Auckland, 1023 Auckland, New Zealand; 2 Gravida National Centre for Growth and Development, The University of Auckland, 1023 Auckland, New Zealand; 3 Department of Anatomy and Medical Imaging, The University of Auckland, 1023 Auckland, New Zealand; 4 Centre for Brain Research, The University of Auckland, 1023 Auckland, New Zealand; 5 Lab Plus, 1023 Auckland, New Zealand; 6 Auckland City Hospital, 1023 Auckland, New Zealand; 7 Department of Pharmacology and Clinical Pharmacology, The University of Auckland, Private Bag 92019, 1142 Auckland, New Zealand

**Keywords:** Inflammation, Blood-brain barrier, Proliferation, Migration

## Abstract

**Background:**

Neuroinflammation and blood-brain barrier (BBB) disruption are common features of many brain disorders, including Alzheimer’s disease, epilepsy, and motor neuron disease. Inflammation is thought to be a driver of BBB breakdown, but the underlying mechanisms for this are unclear. Brain pericytes are critical cells for maintaining the BBB and are immunologically active. We sought to test the hypothesis that inflammation regulates the BBB by altering pericyte biology.

**Methods:**

We exposed primary adult human brain pericytes to chronic interferon-gamma (IFNγ) for 4 days and measured associated functional aspects of pericyte biology. Specifically, we examined the influence of inflammation on platelet-derived growth factor receptor-beta (PDGFRβ) expression and signalling, as well as pericyte proliferation and migration by qRT-PCR, immunocytochemistry, flow cytometry, and western blotting.

**Results:**

Chronic IFNγ treatment had marked effects on pericyte biology most notably through the PDGFRβ, by enhancing agonist (PDGF-BB)-induced receptor phosphorylation, internalization, and subsequent degradation. Functionally, chronic IFNγ prevented PDGF-BB-mediated pericyte proliferation and migration.

**Conclusions:**

Because PDGFRβ is critical for pericyte function and its removal leads to BBB leakage, our results pinpoint a mechanism linking chronic brain inflammation to BBB dysfunction.

**Electronic supplementary material:**

The online version of this article (doi:10.1186/s12974-016-0722-4) contains supplementary material, which is available to authorized users.

## Background

Inflammation and disruption of the blood-brain barrier (BBB) are present in virtually all neurodegenerative diseases, as well as epilepsy, stroke, and traumatic brain injury [1–4]. In Alzheimer’s disease (AD), Huntington’s disease, and Parkinson’s disease (PD), neuroinflammation appears to be an early event in disease progression [5–7]. Most notably, the risk of AD is significantly increased following episodes of an acute inflammatory insult such as infection or with chronic inflammatory conditions such as diabetes [4, 8–11]. Furthermore, inflammation in models of AD, multiple sclerosis (MS), and stroke can induce BBB damage and hence exacerbate and even precede neuropathology [12–14]. In the case of amyotrophic lateral sclerosis, several studies have now shown that blood-spinal cord barrier (BSCB) dysfunction precedes motor neuron damage, and therefore, both the BBB and BSCB have been posed as a potential therapeutic target for early treatment [15, 16]. Furthermore, reparation of the BSCB in a model of motor neuron disease slows disease progression [17].

Therefore, current evidence suggests that inflammation and loss of BBB and BSCB integrity may contribute to disease development as opposed to being a consequence of existing neuropathology [18, 19].

The neurovascular unit is a key functional component of the BBB that is made up of closely connected endothelial cells, pericytes, glia, and neurons [20]. Although previous attention has been focused on other cell types, the scientific community is developing a new appreciation for brain pericytes and their role in neuroinflammatory processes [21]. Brain pericytes can respond to inflammatory signals such as circulating cytokines and convey this information to surrounding cells by way of chemokine and cytokine secretion [22–26]. However, pericytes are also vital to BBB function as a reduction in pericyte coverage causes vascular and barrier defects [27]. In diseases such as diabetic retinopathy, and amyotrophic lateral sclerosis, BBB and BSCB impairment has been linked to pericyte deficiency, while in human AD, BBB impairment is directly correlated with the degree of pericyte dysfunction [28–31]. The coinciding presence of inflammation, BBB breakdown, and pericyte loss in brain disease indicates that inflammation may be one of the drivers of BBB and BSCB leakiness. Although the mechanism for this is not currently understood, we propose that inflammation modulates pericytes specifically and therefore alters the condition of the BBB/BSCB.

One potential mechanism might be the regulation of signalling through the platelet-derived growth factor receptor-beta (PDGFRβ). This is a well-known receptor tyrosine kinase, commonly used as a marker for pericytes, and is crucial to the regulation of survival, proliferation, and migration signals of pericytes [32]. In the central nervous system (CNS), homodimers of platelet-derived growth factor beta subunit (PDGF-BB) are secreted by endothelial cells and bind PDGFRβ on the cell surface of pericytes to promote pericyte vascular coverage in the BBB [33, 34]. Examination of pericyte-deficient animals has revealed that the PDGFRβ signalling pathway is required for pericyte survival and consequently BBB development as well as proper function during adulthood and ageing [27, 35]. Furthermore, previous studies have indicated that PDGFRβ signalling is altered in response to inflammatory signals, although this has not been investigated in human brain pericytes [36, 37].

Since inflammation, pericyte loss and subsequent BBB impairment are widespread in neurodegenerative disorders, identifying how inflammation may modulate pericyte function, and therefore contribute to pathology is of great importance. To study these processes in vitro, we used a model of chronic interferon-gamma (IFNγ)-mediated inflammation and investigated its effects on human brain pericytes [36]. IFNγ is a central component of the inflammatory response in the CNS and can be secreted by microglia, astrocytes, and endothelial cells as well as circulating immune cells [38–42]. This classical inflammatory mediator has been implicated in AD, PD, autoimmune disease, and BBB disruption and provided a suitable system to model a chronic CNS inflammatory environment and its effects on human brain pericytes [41, 43, 44].

## Methods

### Biopsy of human brain tissue

Human brain tissue from both male and female patients was obtained from biopsy at Auckland City Hospital following surgery for intractable temporal lobe epilepsy and approved by the Northern Regional Ethics Committee.

### Human brain cell isolation from epilepsy tissue

Isolation of pericytes from human brain tissue was carried out as previously described [22, 23]. Cells were seeded at 1.0 × 10^6^ for T75 flasks or plated at a density specifically optimized for each set of experiments as detailed below. Cells were cryopreserved in FBS with 5 % dimethyl sulfoxide (DMSO) at a density of 1.0 × 10^6^ cells/mL.

### Cell treatments

#### Model of chronic inflammation

Pericytes were seeded at 3.0 × 10^3^ cells/well or 1.3 × 10^5^ cells/well in either 96-well (for immunocytochemistry) or 6-well plates (for western blot or RNA extraction) in complete media (DMEM/F12 with 10 % FBS and 1 % PSG (penicillin 100 U/ml, streptomycin 100 μg/ml, L-glutamine 0.29 mg/ml)). After 24–48 h of culture, media was replaced with vehicle (0.1 % bovine serum albumin (BSA) in PBS) or IFNγ (R&D Systems (Minneapolis, MN, USA) 285-1F), each diluted 1/100 into cell culture media for a final concentration of 1 ng/mL. Cells were treated every 24 h for a total of 4 days. Twenty-four hours after the fourth treatment, cells were serum starved for 2 h to bring phosphorylation levels to baseline and then treated with vehicle (0.1 % BSA in 4 mM HCl) or PDGF-BB (R&D Systems 220-BB,) diluted 1/100 in cell culture media (final concentration of PDGF-BB was 100 ng/mL) for 30 min. Phosphorylated and total protein expression was measured by western blot, or protein expression and distribution were analysed by immunocytochemistry. Alternatively, for longer treatments (such as proliferation and migration assays), PDGF-BB (10 ng/mL) was used.

### Immunocytochemistry

At endpoint, pericytes were fixed using 4 % paraformaldehyde solution. After cells were washed and permeabilized in PBS with 0.2 % Triton X-100™ (PBS-T), they were incubated with primary antibodies overnight at 4 °C (all antibodies were diluted in goat immunobuffer (1 % goat serum, 0.2 % Triton X-100™, and 0.04 % thiomersal in PBS)). Dilutions and sources of antibodies are listed in Table 1. Plates were washed again in PBS-T, incubated with secondary antibodies for 2–3 h at room temperature, and then rinsed. Nuclei were detected using Hoechst (33258 Sigma, St. Louise, MO, USA).

**Table 1:** Antibodies and dilutions used in this study

	Company	Catalogue number	WB	ICC
Primary antibodies				
PDGFRβ rabbit monoclonal	Cell Signaling	3169	1/1000	1/500
Phospho-PDGFRβ Tyr 751 rabbit monoclonal	Cell Signaling	C63G6	1/1000	
PDGFRβ rabbit monoclonal	Abcam	35270		1/500
αSMA mouse monoclonal	Dako	IS611	1/10	1/4
Akt mouse monoclonal	Cell Signaling	2920	1/2000	
Phospho-Akt S473 rabbit polyclonal	Cell Signaling	9271	1/1000	
Phospho-ERK mouse monoclonal	Santa Cruz	sc-7383	1/500	
PDGF-BB rabbit polyclonal	Abcam	ab23914	1/800	
ERK 1 (K-23) rabbit polyclonal	Santa Cruz	sc-94 rabbit	1/500	
GAPDH mouse monoclonal	Abcam	ab9484	1/1500	
CD140b (PDGFRβ-N-terminal)	AbD Serotec	7460-3104		1/200
Ki67 rabbit polyclonal	Dako	A0047		1/500
^a^CD140b-PE	BD Biosciences	558821		1/5
Secondary antibodies				
Goat anti-rabbit Alexa 488	Life Technologies	A11034		1/500
Goat anti-mouse Alexa 594	Life Technologies	A11032		1/500
Goat anti-mouse IRDye-680LT	LiCOR	926-68020	1/10000	
Goat anti-rabbit IRDye-800CW	LiCOR	926-32211	1/10000	

All antibodies used in this study are listed with the company of origin, the method (*WB* western blot, *ICC* immunocytochemistry), and the dilution used

^a^Used for flow cytometry

### Acquisition and analysis of immunocytochemistry images

Image acquisition was done using the ImageXpress micro XLS™ (Molecular Devices) high-content screening system, housed at the Biomedical Imaging Research Unit, University of Auckland [45]. Images were acquired from micro-well plates using the ×10/0.3 Plan or ×20/0.45 NA CFI Super Plan Fluor ELWD ADM objective lens and Lumencor Spectra X configurable light engine source. Excitation and emission filters used are listed in Table 2.

**Table 2:** Excitation and emission parameters for ImageXpress micro XLS™ high content screening system

Cube	Filters	Lumencor light engine	Ex range	Ex peak	Dichroic	Em range	Em peak
Triple 4	DAPI	UV (380–410)	381–399	390/18	436	445–469	457
Triple 4	FITC	Cyan (460–490)	484–504	494/20	514	518–542	530
Triple 4	TRED	Green (535–600)	561–590	575/25	604	612–643	628
Quad 5	DAPI	UV (380–410)	381–399	390/18	410	419–460	440
Quad 5	FITC	Cyan (460–490)	474–496	485/20	504	507/533	521
Quad 5	CY5	Red (620–750)	644–656	650/13	669	675–723	700

Microscope information for all image acquisition in this study including light source, filters, excitation, and emission range and peaks in nanometer

High-content analysis was then performed with MetaXpress™ version 5.3.04 (Molecular Devices) analysis software. Several algorithms were used to analyse immunocytochemical data including:

- Count nuclei: Cells were counted as positive for Hoechst based on specific user-defined parameters. These parameters included approximate minimum and maximum width (μm) and intensity above local background (grey levels). While intensity settings had to be optimized between experimental plates, the size range for positive cells remained constant. Cells between 7 and 30 μm in width were counted as positive, and this number was then used for total cell counts in each well and site acquired.
- Cell scoring/multi-wavelength cell scoring: These features were used for quantification of Ki67 and EdU. Images were acquired at ×20 magnification. Positive cells were those associated with a nucleus (Hoechst-positive) and stained positively for the protein of interest within user-defined settings for intensity and size based on optimized filter settings. Quantification of results were given as a percentage of cells stained positively for the indicated antibody out of the total number of cells as counted positive by Hoechst. All data from proliferation assays were derived from triplicate wells and nine sites per well.
- Integrated intensity: In some cases where the intensity of the stain was more indicative of overall protein expression, as opposed to the number of cells expressing the protein above a given threshold level, it was more appropriate to quantify the integrated intensity of the stain. The integrated intensity per cell as presented in the text is calculated as previously described [46]. Briefly, a user-defined threshold is set for the intensity of the pixels above background levels and below brightly labelled debris. Parameters for size are also incorporated into this analysis to identify positive cells. The integrated intensity for the staining of interest is then calculated by subtracting the background noise from these values and excluding the debris. This number incorporates both the number of pixels above background, and intensity of those pixels minus background, in addition to the total cell number (as counted by Hoechst-positive cells).
- Transfluor assay: This algorithm is designed to count and measure localized granules or puncta and is derived from the granularity assay used previously [46]. Settings were optimized to detect small puncta of PDGFRβ staining within a user-defined size range and intensity above local background. Total number of puncta in an image was then normalized to total cell number (Hoechst).

For each set of analysis, settings were optimized for each antibody label and parameters were tested on at least four randomly selected images from each plate to ensure quantitation was a representative of actual staining. Internal controls were used to ensure basal settings were consistent between repeated experiments. The parameters set are then applied to all images in a sequence (from a single plate), so that comparisons can be made within an experimental plate. Data is presented from the average of those four images from each replicate well. For all experiments presented here, treatment conditions were done in triplicate, except for the migration assays which had six replicates per condition, per experiment.

### Quantitative RT-PCR

**Fig. 1:**
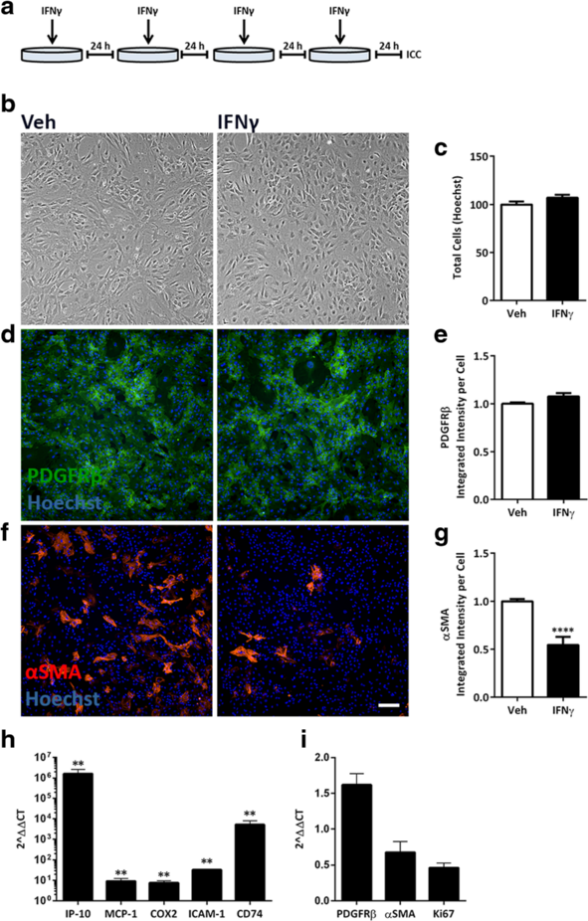
Chronic IFNγ treatment alters αSMA, but not PDGFRβ expression, or cell number in human brain pericytes. **a** Pericytes were treated for four consecutive days (once every 24 h) with either vehicle (Veh) or IFNγ (1 ng/mL) as depicted. **b**, **c** Cells were then fixed and imaged under brightfield (**b**), and total cells counted from Hoechst labelled nuclei (**c**). **d**–**g** Representative images and quantification of PDGFRβ (*green*) (**d**, **e**), or αSMA (*red*) (**f**, **g**) overlaid with Hoechst. *Scale bar*, 100 μm. The integrated intensity of the staining was normalized to cell number (Hoechst) (**c**) and vehicle conditions, quantified from triplicate wells, and plotted as mean ± s.e.m. (*n* = 3) and ****(*p* < 0.0001) (Student’s *t* test). **h**, **i** mRNA from pericytes treated as in **a** was analysed by qRT-PCR. Inflammatory target gene (*IP-10*, *MCP-1*, *COX2*, *ICAM-1*, *CD74*) (**h**) and pericyte marker and proliferation marker gene (*PDGFRβ*, *αSMA and Ki67*) (**i**) expression was normalized to *GAPDH* and plotted as a mean fold change from vehicle (set to 1) (2^ΔΔCT) ± s.e.m. (*n* = 3), **(*p* < 0.01) by a Mann-Whitney, non-parametric test of ΔCT values

Quantitative reverse transcriptase polymerase chain reaction (qRT-PCR) was performed for Fig. 1 as described previously [22, 47]. Alternatively, for Fig. 7, RNA was extracted using the RNAqueous® micro-total RNA isolation kit (Ambion (CA), Life Technologies). cDNA was made from 1.5 μg DNase-1 (Promega)-treated RNA using the Superscript III first-strand synthesis kits (Invitrogen). qRT-PCR was performed using Platinum SYBR Green qPCR SuperMix-UDG with Rox kit (Invitrogen). The level of gene expression was normalized to glyceraldehyde-3-phosphate dehydrogenase (GAPDH) at time zero or untreated conditions using the ΔC_t_ method [48]. The list of primers used is included in Table 3.

**Table 3:** The list of primers used for qRT-PCR in this study

Accession number	Gene		Sequence	Amplicon length
NM_002046.4	GAPDH (h)	Fw	CATGAGAAGTATGACAACAGCCT	113 bp
		Rv	AGTCCTTCCACGATACCAAAGT	
NM_001565.3	IP-10 (CXCL10)	Fw	TGGCACACTAGCCCCACGTT	88 bp
		Rv	TGCTGAGACTGGAGGTTCCTCTGC	
NM_002982.3	MCP-1 (CCL2)	Fw	CAGCCAGATGCAATCAATGCC	190 bp
		Rv	TGGAATCCTGAACCCACTTCT	
NM_000963.2	COX2 (PTGS2)	Fw	AGGGTTGCTGGTGGTAGGAA	76 bp
		Rv	TCTGCCTGCTCTGGTCAATG	
NM_000201.2	ICAM1 (h)	Fw	GAACCAGAGCCAGGAGACAC	84 bp
		Rv	GAGACCTCTGGCTTCGTCAG	
NM_001025159.2	CD74	Fw	GAGTCACTGGAACTGGAGGAC	81 bp
		Rv	CTGCTCTCACATGGGGACTG	
NM_002609.3	PDGFRβ	Fw	CGCAAAGAAAGTGGGCGGCT	101 bp
		Rv	TGCAGGATGGAGCGGATGTGGT	
NM_001141945.2	αSMA	Fw	ACGTGGGTGACGAAGCACAGA	84 bp
		Rv	CGTCCCAGTTGGTGATGATGCC	
NM_001145966.1	Ki67	Fw	AGCGGAAGCTGGACGCAGAA	79 bp
		Rv	TCCAGGGGTTGGGCCTTTTCCT	

### Confocal microscopy

Cells were seeded directly onto #1.5 glass coverslips (Menzel Gläser) in 48-well plates. Twenty-four hours later, cells were treated as above for the chronic cytokine model. After 96 h, cells were serum starved for 2 h and then treated with PDGF-BB (100 ng/mL) or vehicle for 30 min to internalize PDGFRβ. Cells were then fixed with 4 % PFA and processed as above for immunocytochemistry with a PDGFRβ antibody and Hoechst. Coverslips were mounted onto slides using Dako fluorescent mounting media. All confocal images were recorded using a Zeiss LSM710 inverted confocal microscope with a ×63 oil immersion lens (NA 1.4) at 0.32-μm slices. Orthogonal projections were generated using the Fiji plugin for ImageJ software version 1.47k from the National Institute of Health (NIH).

### Live labelling of plasma membrane PDGFRβ

Membrane PDGFRβ was detected using live labelling as previously described with modifications [49]. At endpoint, plated cells were taken onto ice for 90 s, media was removed, and cells were washed with serum-free DMEM/F12 with 5 mg/mL BSA (SFM/BSA). Antibody against the extracellular N-terminus of PDGFRβ (CD140b) was diluted 1/200 into SFM/BSA and added to desired wells. The plate was then incubated at room temperature for 30 min. Following antibody incubation, cells were washed in SFM/BSA, fixed with 4 % PFA for 15 min, and permeabilized with PBS-T for 20 min. Co-labelling and image acquisition was then performed as above.

### Flow cytometry for cell surface PDGFRβ

At endpoint, cells were washed in PBS, incubated with Accutase (A1110501 ThermoFisher) at 37 °C for 5 min, and gently triturated to produce a single-cell suspension. An equal volume of complete media was added to stop enzymatic activity, and cells were centrifuged at 160×*g* for 5 min. The supernatant was discarded, and cells were resuspended in FACS buffer (1 % FBS in PBS). Cells were incubated with 7-aminoactinomycin (7-AAD) (1:20 dilution; 51-68981E, BD Biosciences CA, USA) and CD140b-PE (1:5 dilution; PE mouse anti-human CD140b-PE 558821, BD Biosciences) for 15 min on ice. Cells were centrifuged at 160×*g* for 5 min at 4 °C, the supernatant was discarded, and cells were resuspended in FACS buffer. Samples were run on an Accuri C6 flow cytometer (BD Biosciences), and 8000 viable cells were gated based on forward and side scatter and 7-AAD exclusion. Analysis of flow cytometry data was performed using FlowJo software (v 7.6.5). Mean fluorescence intensity of CD140b cell surface expression was determined from three independent cases.

### Cell proliferation, viability, and migration assays

To measure PDGF-BB-induced cell growth, pericytes were cultured and treated as above for the chronic inflammation model with additional treatments outlined below.

#### Cell proliferation assay

After 48 h of cytokine treatment, either vehicle (0.1 % BSA in 4 mM HCl) or PDGF-BB diluted 1/100 was added to measure PDGF-BB-induced cell proliferation (final concentration of PDGF-BB was 10 ng/mL). Proliferation was measured by 5-ethynyl-2′-deoxyuridin (EdU) with Click-iT®Assay Kit (Life Technologies C10340) according to the manufacturer’s instructions and Ki67 immunocytochemical labelling. Briefly, EdU (5 μM) was added 24 h prior to endpoint and cells incubated for a further 24 h. Cells were fixed with 4 % PFA for 15 min at room temperature. Cells were rinsed with 3 % BSA in PBS and then permeabilized with 0.5 % Triton X-100 in PBS for 20 min at room temperature. Cells were washed twice with 3 % BSA in PBS, and then, EdU reaction cocktail was added for 30 min at room temperature protected from light. Cells were then washed once more with 3 % BSA in PBS and labelled with a Ki67 antibody as described above for immunocytochemistry.

#### AlamarBlue® assay

AlamarBlue® (AbD Serotec BUF012B) was used as a measure of cell health and metabolism. The AlamarBlue® reagent is an oxidation-reduction indicator that produces fluorescence when reduced by media metabolizing cells. AlamarBlue® reagent was added to wells treated as above at a 1/10 dilution, and cells were incubated at 37 °C, 5 % CO_2_ for 1 h prior to endpoint of experiment. Fluorescence was measured using the FLUOStar Optima plate reader (BMG LABTECH) with an excitation of 544 nm and emission of 590 nm. Controls of media alone (no cells) with AlamarBlue® were included, and fluorescence values were used as baseline. Data are presented as mean % AlamarBlue® metabolized per cell and normalized to vehicle controls ± standard error of the mean (s.e.m.).

#### LDH assay

Lactate dehydrogenase (LDH) assay (Roche) was performed as per manufacturer’s instructions. Briefly, at endpoint, cell media from experimental wells as well as controls (cells lysed with Triton X-100™ to obtain 100 % lysis) was transferred to a new cell culture plate and centrifuged at 250×*g*. The supernatant was transferred to a new cell culture plate containing an equal volume of LDH reagent mix and incubated in the dark for 30 min at room temperature. Absorbance was measured at 492 nm from triplicate wells and three cases. Data were normalized to 100 % lysed wells and plotted as mean ± s.e.m.

#### Migration assay

Cells were treated as above for proliferation assay. At the 48-h time point, cells were scratched down the middle of the well with a sterile p200 pipette tip. Cells were washed with complete media to remove unattached debris, and media containing vehicle, or PDGF-BB, diluted 1/100 was added to wells for a further 48 h (final concentration was 10 ng/mL). Chronic cytokine treatment was continued every 24 h for a total of 96 h. Cells were then rinsed and fixed with 4 % PFA for 15 min. Cells were stained by adding Coomassie Blue (0.25 %) in 40 % ethanol and 10 % acetic acid for 30 min at room temperature. Stain was removed, and wells were allowed to dry. Images were acquired as above for immunocytochemistry with modifications; wells were acquired using bright field at ×4 magnification to obtain the scratch area. Cells that had migrated into the scratch area were quantified manually using ImageJ version 1.47k from NIH, using the Cell Counter plugin [50]. Each experiment was counted by two individuals blinded to treatment conditions.

### Western blotting

At endpoint, pericytes were rinsed with PBS and scraped into Eppendorf tubes. Cells were centrifuged, and pellet was resuspended in lysis buffer (25 mM Tris-HCl pH 7.5, 150 mM NaCl, 50 mM NaF, 0.5 mM EDTA pH 8, 0.5 % Triton-X 100™, 5 mM β-glycerophosphate, with fresh 1 mM DTT, 1 mM PMSF, 1 mM Na_3_VO_4_). Of the total protein, 20 % from one well of a six-well plate was diluted 1:1 in 2× Laemmli buffer (125 mM Tris-HCl, pH 6.8, 5 % glycerol, 4 % sodium dodecyl sulphate (SDS), 0.2 % bromophenol blue) and separated on 4–12 % pre-cast gels (Life Technologies) by SDS-polyacrylamide gel electrophoresis.

Fluorescent westerns were carried out as previously described [22]. Briefly, proteins were transferred to polyvinylidene difluoride (PVDF) membranes (Millipore (Billerica, MA, USA) IPFL00010 Immobilon-FL 0.45 mm) for optimal fluorescence signal and blocked in Odyssey® Blocking Buffer (Li-COR (NE, USA) 927-40000) diluted 1:1 in Tris-buffered saline with 0.1 % Tween®-20 (TBS-T), for 1 h at room temperature. Membranes were incubated with primary antibodies (Table 1) diluted in Odyssey® Blocking Buffer and TBS-T (1:1) overnight at 4 °C. Membranes were incubated with secondary antibodies (Li-COR, Table 1) diluted in Odyssey® Blocking Buffer and TBS-T (1:1) with 0.1 % Tween®-20 and 0.02 % SDS for 2 h at room temperature. Images were captured using a Li-COR Odyssey FC® imaging system, and band intensity was quantified using Image Studio™ Lite (Ver 5.0). Fluorescent images were converted to greyscale, and inverted using Adobe Photoshop. Some proteins were analysed on parallel blots to avoid the need to strip the membranes; however, all quantification was completed on intensities from the same blots.

### Statistical analysis

Each experiment was repeated on three individual cases, i.e. pericytes from separate donors, and normalized data were combined and presented as the mean ± s.e.m. unless otherwise stated. GraphPad Prism (Ver 6) for Windows was used for statistical analysis. Data were tested for normal distribution using D’Agostino and Pearson *omnibus* test for normality. Data that were normally distributed was then analysed with either two-tailed student’s *t* test or one-way analysis of variance (ANOVA) with Dunnett’s post hoc test for multiple comparisons. If data were not normally distributed, then the non-parametic Kruskal-Wallis test was used followed by Dunn’s multiple comparison tests to determine significance. Alternatively, for qRT-PCR data, the Mann-Whitney rank-sum test was used to analyse the ΔCT values. Two-way ANOVA was used when comparing groups of independent variables, with Tukey’s multiple comparison tests. Significance from control conditions is indicated by *(*p* < 0.05), **(*p* < 0.01), ***(*p* < 0.001), and ****(*p* < 0.0001).

## Results

### Chronic IFNγ treatment modulates pericyte morphology and phenotype

Pericytes exposed to IFNγ were analysed for their growth and cell marker expression (Fig. 1a). IFNγ-treated cells appeared to grow at a similar rate to vehicle-treated cells, they adopted a more spread-out, flat, cobblestone-like pattern (Fig. 1b), and total cell number was not changed after 4 days of IFNγ treatment (Fig. 1c).

The expression of pericyte markers PDGFRβ and alpha smooth muscle actin (αSMA) were also examined by immunocytochemistry. Integrated intensity was used as a measure of overall expression of either αSMA or PDGFRβ staining employing a previously published algorithm [46]. IFNγ treatment did not significantly alter the expression of PDGFRβ (Fig. 1d, e) but considerably reduced the staining intensity of αSMA (Fig. 1f, g). Consistent with previous reports of tumour necrosis factor alpha (TNFα) and interleukin-1beta (IL-1β) treatment in pericytes, both PDGFRβ and αSMA protein expression were reduced after chronic treatment of either cytokine [51] (Additional file 1: Figure S1).

We have previously shown that inflammatory cues alter gene expression in human brain pericytes [22, 23]. Therefore, using gene hits obtained from a previous microarray study [22], we examined gene expression changes in pericytes under chronic IFNγ conditions. Inflammatory response genes interferon-inducible protein-10 (*IP-10*), monocyte chemotactic protein-1 (*MCP-1*), cyclooxygenase 2 (*COX2*), intracellular adhesion molecules (*ICAM-1*), and cluster of differentiation (*CD74*) remained elevated after 96 h of IFNγ treatment (Fig. 1h). We also measured messenger RNA (mRNA) expression of *PDGFRβ*, *αSMA* and *Ki67* (Fig. 1i). Interestingly, although mRNA data matched observations for protein levels of αSMA, we saw a discrepancy in PDGFRβ mRNA and protein expression. Despite having seen no change at the protein level, chronic IFNγ conditions caused a 1.5-fold increase in *PDGFRβ* transcripts compared to controls, which was consistent across three separate cases. In addition, there was a consistent trend of a decrease in *Ki67* after IFNγ treatment which suggested an inhibitory effect on cell proliferation.

### PDGFRβ signalling in adult human brain pericytes

**Fig. 2:**
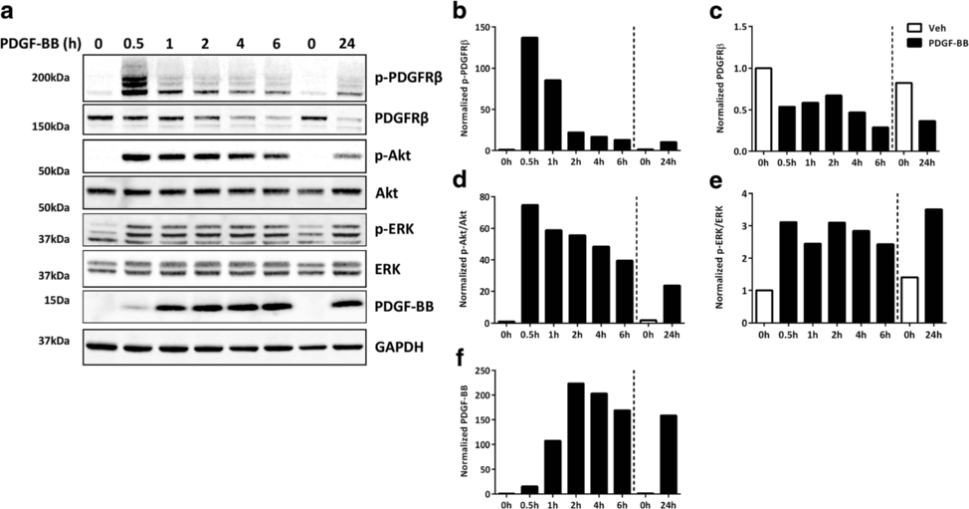
Human brain pericytes respond to PDGF-BB signals in vitro*.* **a** Pericytes were serum starved for 2 h, then treated with vehicle (lanes 1 and 7) or PDGF-BB (100 ng/mL) (lanes 2–6, and 8) for the indicated times, and analysed by SDS-PAGE. Representative blots from three cases are shown. **b** Blots from **a** were analysed and quantified with Image Studio™. Phosphorylated PDGFRβ (Tyr751) (p-PDGFRβ) (**b**), PDGFRβ (**c**), and PDGF-BB (**f**) were normalized to GAPDH; phosphorylated Akt (Ser473) (p-Akt) (**d**) and phosphorylated ERK (Tyr204) (p-ERK) (**e**) were normalized to total Akt and ERK, respectively. **Bottom band in pERK blot is GAPDH

PDGFRβ plays an important role in pericyte function. We therefore first sought to determine the normal physiological response of human brain pericytes to PDGF-BB in the absence of IFNγ by measuring activation of downstream signalling proteins. Pericytes cultured as previously described [22] were serum starved for 2 h and then treated with vehicle or PDGF-BB (10 ng/mL) for 30 min up to 24 h. Tyrosine 751 (Tyr751) is known to be the major residue of the PDGFRβ that is phosphorylated in response to PDGF-BB stimulation and responsible for association of the receptor with phosphotidylinositol-3-kinase (PI3K) [52]. Thus, phosphorylation of PDGFRβ at Tyr751 was used as marker of receptor activation. Activation of the PDGFRβ was detected after 30 min of PDGF-BB treatment and gradually decreased over the 24-h treatment time (Fig. 2a, b and Additional file 2: Figure S2). This observation was consistent with previous reports of commercial human brain pericytes [53]. Examination of downstream pathway activation demonstrated elevated levels of phosphorylated Akt and extracellular signal-regulated kinase (ERK) following PDGF-BB treatment, which was maintained up to 24 h (Fig. 2a, d, e) and was consistent with studies in other cell types [54, 55]. In addition, levels of PDGFRβ declined with exposure to the ligand (Fig. 2a, c), suggesting an agonist-induced downregulation. We also detected elevated levels of PDGF-BB in cell lysates, from 1–24 h (Fig. 2a, f). This is most likely due to the detection of exogenous PDGF-BB internalizing with the PDGFRβ.

### Chronic IFNγ enhances PDGF-BB-induced PDGFRβ phosphorylation and internalization

**Fig. 3:**
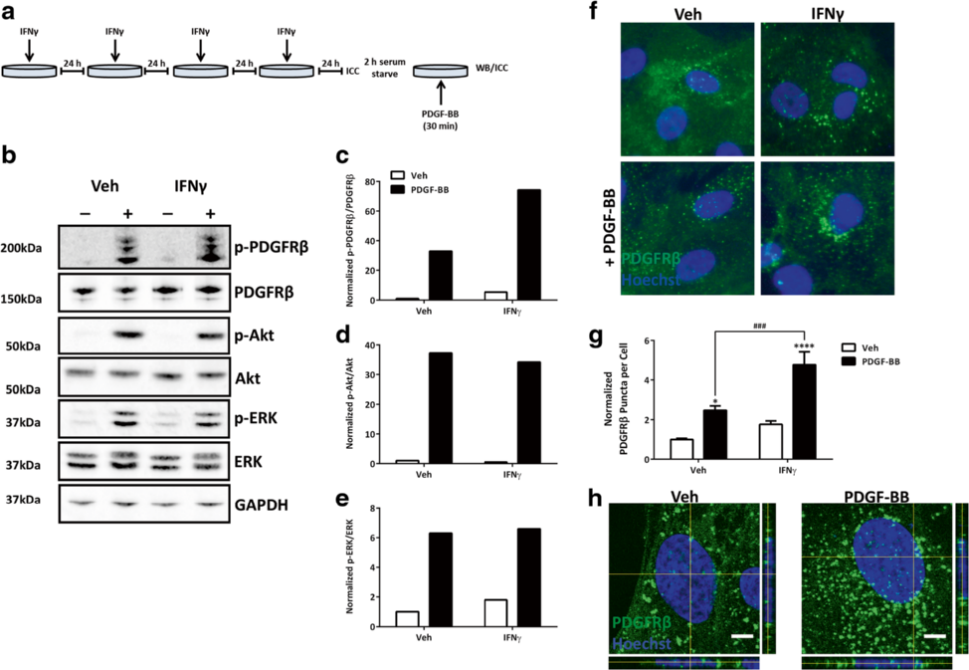
Chronic IFNγ treatment increases PDGFRβ phosphorylation and internalization. **a** Pericytes were treated for four consecutive days (once every 24 h) with either vehicle (*Veh*) or IFNγ (1 ng/mL). After 96 h total treatment, cells were serum starved for 2 h and then treated with vehicle (−) or PDGF-BB (100 ng/mL) for 30 min as depicted. **b** Representative western blots of treated pericytes (*n* = 3). **c**–**e** Bands were quantified with Image Studio™ and normalized to vehicle control. p-PDGFRβ (**c**) was normalized to total PDGFRβ, and p-Akt (**d**) and p-ERK (**e**) were normalized to total Akt and ERK, respectively. **f** Representative images of pericytes treated as in **a** showing PDGFRβ (*green*) and Hoechst (*blue*), *scale bar*, 10 μm. **g** PDGFRβ puncta in **f** were quantified using MetaXpress™ software and normalized to cell number and vehicle control and plotted as mean ± s.e.m. (*n* = 3), ****(*p* < 0.0001), ^###^(*p* < 0.001), *(*p* < 0.05) (two-way ANOVA). **h** Cellular localization of PDGFRβ (*green*) and nuclei (*blue*) in pericytes treated with vehicle (*Veh*) or PDGF-BB (100 ng/mL) for 30 min as above using confocal microscopy. *Scale bar*, 5 μm

Since PDGFRβ plays such an important role in pericyte survival, and previous work has shown cytokines to alter PDGF signalling in other cell types [37, 51], we were interested in whether chronic IFNγ could influence PDGFRβ activation. Pericytes were treated with IFNγ for 4 days as described in the methods, and the PDGF-BB-induced phosphorylation status of PDGFRβ (Tyr751) was examined by western blot (Fig. 3a). As expected, there was no detectable PDGFRβ phosphorylation under serum-starved vehicle-treated conditions. Treatment with PDGF-BB (100 ng/mL) for 30 min induced phosphorylation of PDGFRβ at Tyr751 (Fig. 3b). Interestingly, the phosphorylation of PDGFRβ in response to PDGF-BB was greater in pericytes chronically treated with IFNγ than controls (Fig. 3b, c and Additional file 3: Figure S3). Since it has been well-documented that PDGFRβ activation can act through both the PI3K-Akt, as well as mitogen-activated protein kinase (MAPK)-ERK pathways [34, 56], we were interested in whether heightened PDGFRβ activation induced by IFNγ would also be reflected in the downstream pathways. However, western blot results did not indicate any changes with Akt or ERK activation under the inflammatory conditions tested (Fig. 3b, d, e and Additional file 3: Figure S3).

Characteristic PDGF signalling involves binding of the ligand to the receptor, followed by dimerization, autophosphorylation, and internalization [57]. Additionally, it has been shown that PDGFRβ internalization can occur independently of receptor phosphorylation [58]. We therefore examined PDGF-BB-induced receptor internalization by immunocytochemistry. Upon close examination of PDGFRβ staining, we observed that PDGF-BB treatment increased the number of puncta in the cell, which is indicative of receptor internalization (Fig. 3f) [37]. The number of puncta per cell was quantified using the transfluor algorithm within the MetaXpress™ image analysis software and normalized to vehicle-treated controls (Fig. 3g). Treatment of pericytes with PDGF-BB (100 ng/mL) for 30 min produced an increased number of PDGFRβ puncta in control conditions. However, cells treated chronically with IFNγ showed a marginal increase in puncta per cell under basal (vehicle treated) conditions, which was enhanced under ligand (PDGF-BB)-stimulated conditions. This observation suggested that IFNγ increased PDGF-BB-induced PDGFRβ internalization. There was, however, no change in PDGFRβ puncta with TNFα or IL-1β treatment in pericytes (Additional file 4: Figure S4). We therefore focused our investigations on the mechanisms and outcomes of IFNγ-induced changes in PDGFRβ signalling in pericytes.

In order to confirm that PDGFRβ puncta represented internalized receptor, we performed confocal microscopy on pericytes stimulated with vehicle or PDGF-BB for 30 min. Confocal analysis confirmed the localization of the PDGFRβ puncta inside the cell (Fig. 3h).

### Membrane PDGFRβ is increased by IFNγ treatment

**Fig. 4:**
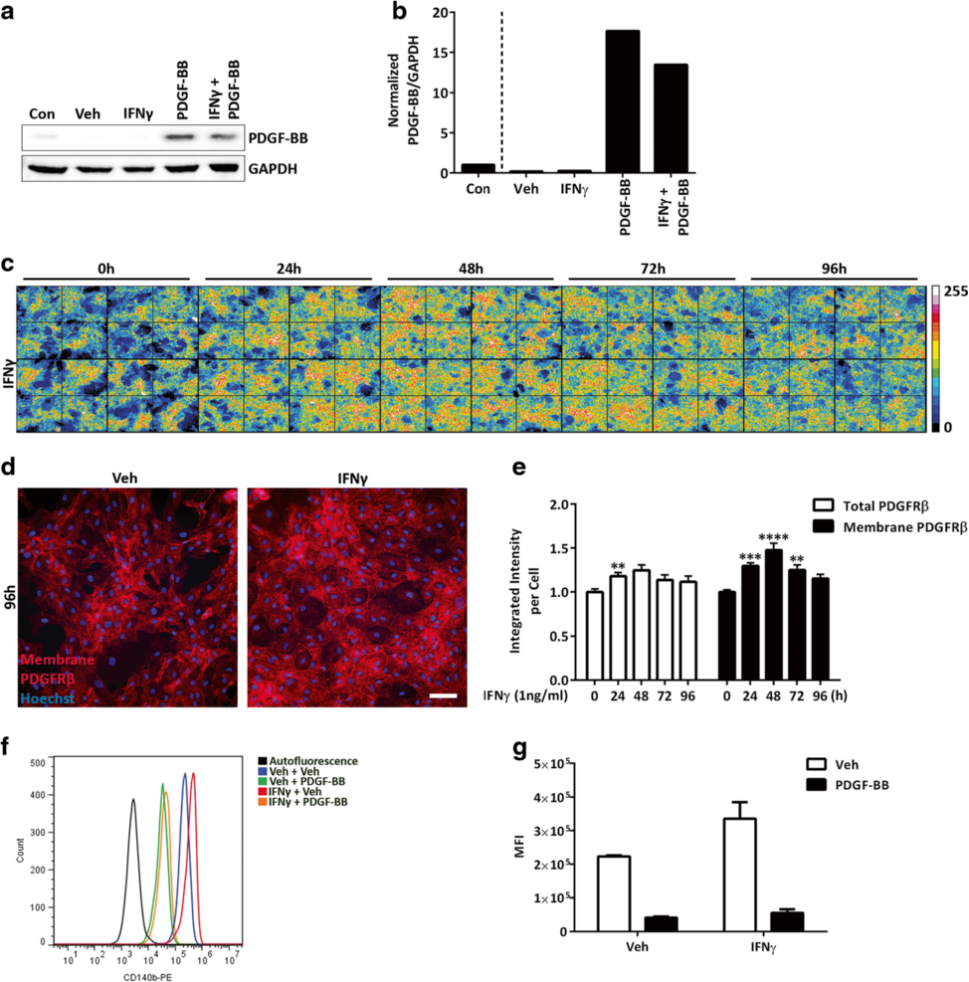
Chronic IFNγ increases PDGFRβ membrane expression. **a**, **b** Detection of PDGF-BB in pericyte conditioned media. Human brain pericytes at 90 % confluence were left untreated (control (*Con*)) or serum starved for 2 h and then treated with vehicle, IFNγ (1 ng/mL), PDGF-BB (100 ng/mL), or both IFNγ and PDGF-BB for 24 h. Lysates were collected for western blot analysis with the indicated antibodies, and a representative blot is shown (*n* = 2) (**a**) Bands were quantified with Image Studio™ software and intensity was normalized to vehicle control (**b**); PDGF-BB was normalized to GAPDH. **c**–**e** Pericytes were treated for 0, 24, 48, 72, or 96 h (more cytokines added once every 24 h to appropriate wells) with vehicle (*Veh*) or IFNγ (1 ng/mL). **c** Grey value intensities of PDGFRβ membrane staining are depicted in a pseudo-colour image according to the legend (*right*). **d** Representative images of membrane PDGFRβ (*red*) and Hoechst (*blue*) in pericytes after 96 h of vehicle or IFNγ treatment. *Scale bar*, 100 μm. **e** Quantification of total PDGFRβ (*white bars*) and membrane PDGFRβ (*black bars*) staining intensity per cell was normalized to 0 h time point, plotted as mean ± s.e.m. (*n* = 3), ****(*p* < 0.0001), ***(*p* < 0.001), **(*p* < 0.01) (ANOVA). **f**, **g** Pericytes were treated for four consecutive days (once every 24 h) with either vehicle (*Veh*) or IFNγ (1 ng/mL). After 96 h total treatment, cells were serum starved for 2 h and then treated with vehicle (*Veh*) or PDGF-BB (100 ng/mL) for 30 min. Surface PDGFRβ expression was analysed using flow cytometry, and a representative plot is shown (**f**). Mean fluorescence intensity (*MFI*) of cell surface PDGFRβ is plotted as mean ± s.e.m. (*n* = 3) (**g**)

We wondered whether the mechanism for enhanced PDGF-BB-induced PDGFRβ internalization under chronic IFNγ-treated conditions may involve IFNγ enhancing PDGF-BB production and therefore PDGFRβ pathway activity. Western blot analysis of pericytes after 24 h of IFNγ treatment, however, did not show a detectable increase in PDGF-BB in the lysate (Fig. 4a, b). Another possible mechanism is that IFNγ may induce the recruitment of PDGFRβ to the cell surface and thus increase the ligand-accessible membrane pool. To address this possibility, we performed live labelling of PDGFRβ to detect membrane bound PDGFRβ and compared this with total staining of permeabilized cells. Interestingly, there was a more extensive increase in cell surface expression of PDGFRβ under chronic IFNγ treatment, and this continued from 24 to 72 h (Fig. 4c). Observations of the pattern of PDGFRβ membrane staining also showed an increase after 96 h of IFNγ treatment (Fig. 4d). Quantification of the staining intensity of membrane versus total PDGFRβ expression was consistent with an increase of membrane PDGFRβ over time with IFNγ treatment (Fig. 4e). In contrast, TNFα and IL-1β treatment decreased both total and membrane PDGFRβ expression over time in a similar manner (Additional file 5: Figure S5), indicating that the re-distribution of PDGFRβ to the cell surface was specific to IFNγ and not a general pro-inflammatory response.

Flow cytometry analysis of membrane PDGFRβ confirmed immunocytochemistry data. Pericytes treated for 30 min with PDGF-BB showed less surface PDGFRβ, a result consistent with internalization of the receptor (Fig. 4f, g). Furthermore, surface PDGFRβ detection by flow cytometry was increased when pericytes were pre-treated for 48 h with IFNγ. Ligand-stimulated internalization of PDGFRβ in the presence of IFNγ was retained as demonstrated by similar reduction in surface PDGFRβ detection following PDGF-BB treatment. Thus, chronic IFNγ increases the membrane-bound pool of the PDGFRβ and therefore the amount of receptor available for stimulation with the PDGF-BB ligand.

### IFNγ blocks PDGF-BB-dependent pericyte proliferation

**Fig. 5:**
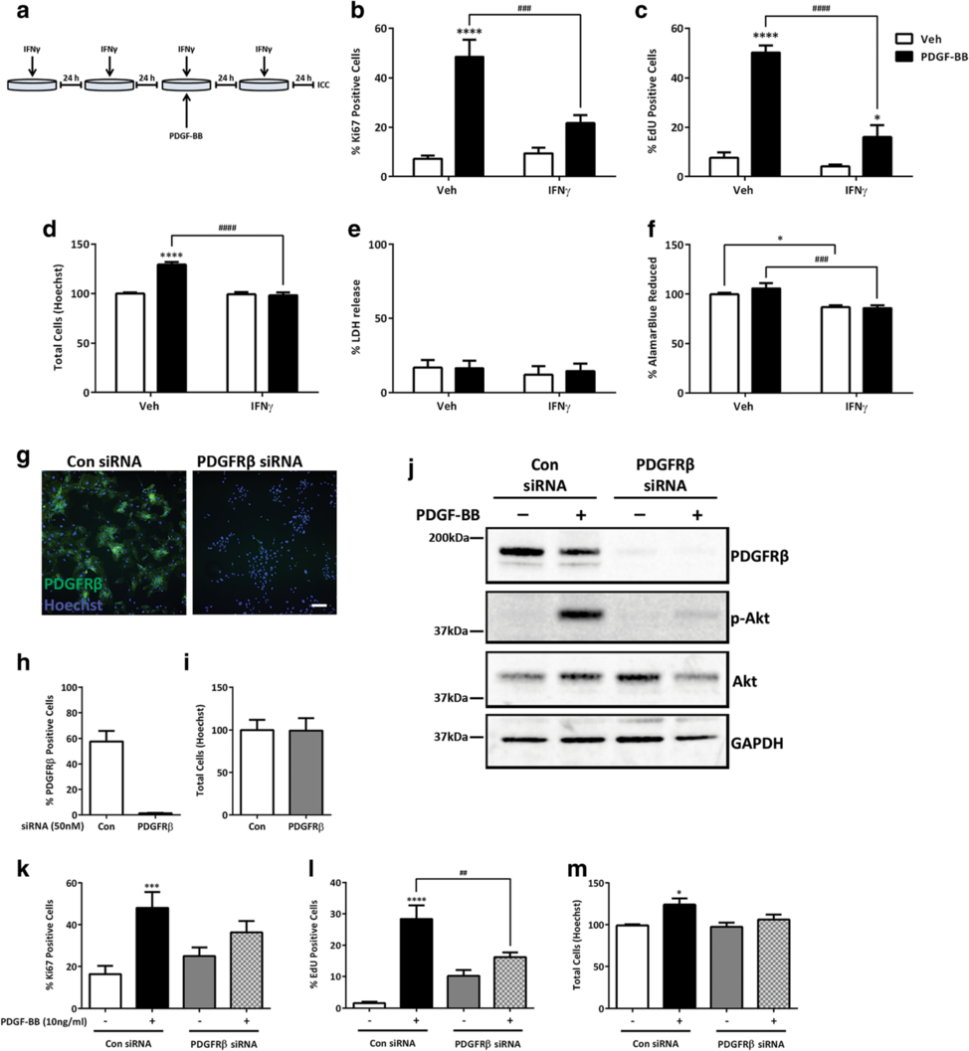
Chronic IFNγ treatment and PDGFRβ knockdown blocks PDGF-BB-induced proliferation in pericytes. **a** Pericytes were treated for four consecutive days (once every 24 h) with either vehicle (*Veh*) or IFNγ (1 ng/mL). **b**, **c** After 48 h of cytokine treatment, cells were treated with either vehicle or PDGF-BB (10 ng/mL) to measure the PDGF-induced proliferative response. This was done in two ways: after 96 h total treatment, cells were fixed, labelled with a Ki67 antibody and Hoechst (**b**); alternatively, EdU was added to measure cell proliferation over the final 24 h of the experiment (**c**). Positive cells of the total cells measured by Hoechst (**d**) were quantified and plotted as mean ± s.e.m. (*n* = 3), ****, ^####^(*p* < 0.0001), ***(*p* < 0.001), *(*p* < 0.05) from a two-way ANOVA. **e**, **f** LDH release (**e**) and AlamarBlue reduction (**f**) were also measured as cell death and cell health outputs, respectively, from the above proliferation experiments. **g** Knockdown of PDGFRβ in pericytes with siRNA after 96 h immunolabelled for PDGFRβ (*green*) and Hoechst (*blue*), *scale bar*, 100 μm. **h**, **i** Quantification of pericytes positive for PDGFRβ after siRNA knockdown. Percent positive for PDGFRβ (**h**) of the total cells measured by Hoechst (**i**), mean (per well) ± s.e.m. (*n* = 1). **j** Representative western blot of PDGF-BB response in PDGFRβ deficient pericytes (*n* = 2). **k**–**m** Proliferation response to PDGF-BB in PDGFRβ deficient pericytes after 48 h. Ki67 (**k**), EdU (**l**) positive, and total cells (**m**) mean ± s.e.m. (*n* = 5) were plotted and analysed with two-way ANOVA, ****(*p* < 0.0001), ^###^(*p* < 0.001), ***(*p* < 0.001), and *(*p* < 0.05)

The importance of PDGF-BB-dependent signalling for pericyte proliferation and migration in the formation of new blood vessels of the BBB has been well-documented in animal models [33]. However, it is currently unknown what role PDGFRβ plays in adult human brain pericytes and how these particular cells may be affected by a pro-inflammatory environment. Past observations of cytokine effects on PDGF-induced proliferation and migration have been conflicting depending on the cell types studied [56]. We therefore analysed PDGF-BB-induced cell proliferation in human brain pericytes (Fig. 5a). Under vehicle pre-treated conditions, we observed an increase in both Ki67 (Fig. 5b)- and EdU (Fig. 5c)-positive cells, as well an increase in total pericyte counts after 48 h of PDGF-BB treatment (Fig. 5d). However, when these parameters were measured under chronic IFNγ conditions, there was a dramatic blunted proliferative response to PDGF-BB when measured by either Ki67- or EdU-positive cells or total cells (Fig. 5b–d). The observed decrease in proliferation did not appear to be related to cell metabolism or cell death as measured by AlamarBlue® or LDH, respectively, as this was unchanged in PDGF-BB compared to vehicle controls (Fig. 5e, f). However, there was a significant decrease in cell metabolism with IFNγ treatment that was not dependent on PDGF-BB (Fig. 5f). Thus, chronic IFNγ decreases cell metabolism and blocks PDGF-BB-dependent proliferative effects in human brain pericytes. Alternatively, TNFα or IL-1β treatment did not significantly alter PDGF-BB-induced proliferation in pericytes (Additional file 6: Figure S6).

To determine whether PDGFRβ is responsible for the proliferative action of PDGF-BB on human brain pericytes, we used small interfering RNA (siRNA) to knockdown receptor expression (Fig. 5g). PDGFRβ siRNA reduced PDGFRβ expression to ≤5 % in human brain pericytes without affecting cell number (Fig. 5h, i). Knockdown of PDGFRβ dramatically inhibited PDGF-BB-induced Akt phosphorylation (Fig. 5j). Reduction in PDGFRβ expression also resulted in markedly reduced proliferation in response to PDGF-BB stimulation similar to IFNγ treatment conditions as measured by Ki67, EdU, and total cell counts (Fig. 5k, l, m). These combined data indicate that PDGFRβ mediates the PDGF-BB-induced proliferation response in human brain pericytes and suggests that IFNγ is acting through inhibition of the PDGF signalling pathway to block cell proliferation signals. Interestingly, we did not detect any loss of viability of human brain pericytes depleted of PDGFRβ in contrast to animal knockout studies [35] (Fig. 5m).

### Migration of human brain pericytes is blocked by IFNγ

**Fig. 6:**
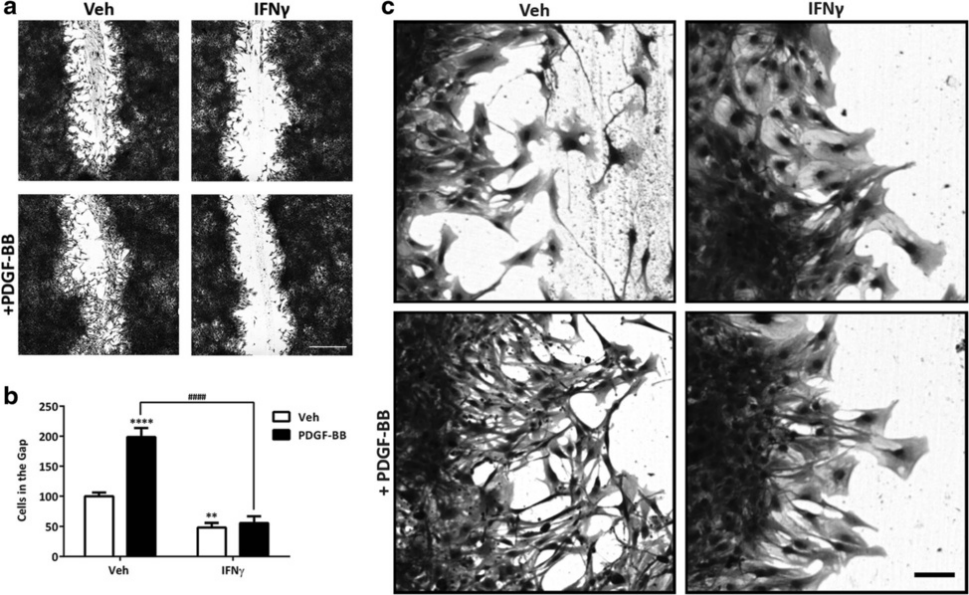
IFNγ blocks pericyte migration independently of PDGF-BB. **a**, **b** Cell migration was measured by scratching wells after 48 h of IFNγ (1 ng/mL) treatment and measuring the number of cells that moved into the gap area after a further 48 h with vehicle or PDGF-BB (10 ng/mL) treatment. Representative images of Coomassie-stained pericytes are presented in **a**, and manual counts of cells in the gap area (normalized to vehicle-treated condition), plotted as mean ± s.e.m. (*n* = 3) ^####^, ****(*p* < 0.0001), **(*p* < 0.01), (two-way ANOVA), are presented in **b** *Scale*, 500 μm. **c** Magnified view of pericytes from migrating edge of scratch wound from conditions in **a** *Scale bar*, 100 μm

To further study the actions of IFNγ on human brain pericytes, we investigated pericyte migration, which is an important property of these cells [33, 53]. We assessed the ability of pericytes to migrate in a scratch wound assay (refer to Fig. 6a). After 48 h of PDGF-BB treatment, we detected an increase in migration of pericytes into the wound area compared to controls (Fig. 6a, b). In congruence with proliferation data, under chronic IFNγ conditions, PDGF-induced movement of pericytes into the wound area was greatly reduced. However, in contrast to the effect on proliferation, IFNγ also caused a significant inhibition of migration in the absence of PDGF-BB treatment. These data would indicate that IFNγ can modulate pericyte function by both PDGF-dependent and independent means. Closer observations of morphology of migrating cells demonstrated that under control conditions, pericytes were elongated and extended out towards the gap area, whereas when exposed to IFNγ, cells adapted a more polygonal-like structure (Fig. 6c).

### IFNγ inhibits PDGF-BB-induced PDGFRβ re-expression

Although our results from proliferation and migration assays were consistent with previous reports of cell growth inhibition by IFNγ, the increase in PDGFRβ membrane expression, phosphorylation, and internalization was not consistent with this effect. It has been documented in the literature that receptor internalization can be used by the cell as a regulatory mechanism to switch off a particular pathway, and avoid chronic signal activation; indeed, this has been observed for PDGFRβ pathway regulation [59]. Following internalization, receptor tyrosine kinases are known to recycle back to the cell surface or to be degraded, each promoting distinct cellular outputs. The predominant method of PDGFRβ regulation is through receptor ubiquitination and subsequent degradation by lysosomes and proteasomes [60]. As shown in Fig. 2b, levels of PDGFRβ declined significantly with exposure to the PDGF-BB (maximally at 24 h after ligand addition), suggesting that this mechanism of receptor degradation might also be operating in human brain pericytes. Hence, it was possible that despite increasing cell surface expression of PDGFRβ, chronic IFNγ treatment had a negative influence on signalling outputs by promoting the internalization and degradation of PDGFRβ after ligand stimulation.

**Fig. 7:**
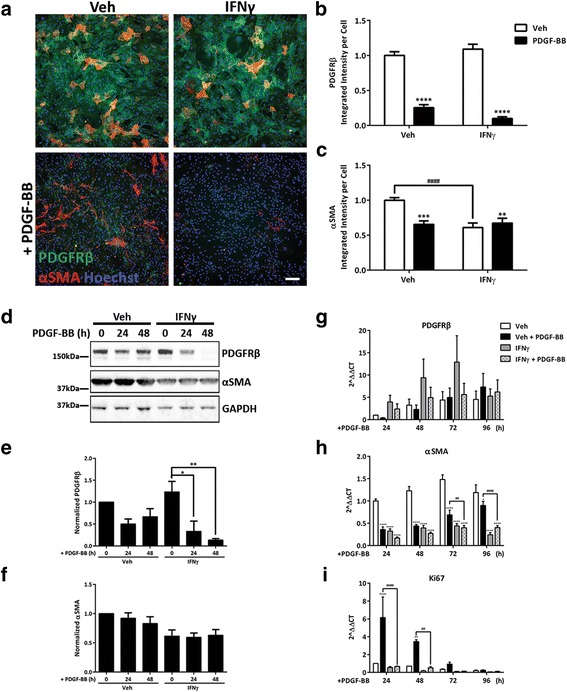
Chronic IFNγ treatment reduces PDGFRβ re-synthesis following ligand-stimulated degradation. **a**–**c** Pericytes were treated for four consecutive days (once every 24 h) with either vehicle (*Veh*) or IFNγ (1 ng/mL); the final 48 h was in the presence of either vehicle or PDGF-BB (10 ng/mL), with representative images of PDGFRβ (*green*), αSMA (*red*), and Hoechst (*blue*) (**a**) *Scale bar*, 100 μm. Quantification of PDGFRβ (**b**) and αSMA (**c**) staining, mean ± s.e.m. (*n* = 3), ^####^, ****(*p* < 0.0001), ***(*p* < 0.001), **(*p* < 0.01) (two-way ANOVA). **d**–**f** Pericytes were treated for three or four consecutive days (once every 24 h) with either vehicle (*Veh*) or IFNγ (1 ng/mL). After 48 h, cells were treated with PDGF-BB (10 ng/mL) for either 24 or 48 h. Representative blots of PDGFRβ, αSMA, and GAPDH (**d**) Quantification of band intensity of PDGFRβ (**e**) and αSMA (**f**), both normalized to GAPDH, mean ± s.e.m. (*n* = 3), **(*p* < 0.01), *(*p* < 0.05) (two-way ANOVA). **g**–**i** Pericytes were pre-treated for 48 h (once every 24 h) with either vehicle or IFNγ (1 ng/mL) and then treated with PDGF-BB (10 ng/mL) for 24, 48, 72, or 96 h (with IFNγ being added every 24 h throughout). qRT-PCR analysis of *PDGFRβ* (**g**), *αSMA* (**h**), and *Ki67* (**i**) transcripts was normalized to vehicle treatment and plotted as mean ± s.e.m. (*n* = 3), ^####^, ****(*p* < 0.0001), ^##^, **(*p* < 0.01), *(*p* < 0.05) (two-way ANOVA)

We therefore examined PDGFRβ expression by immunocytochemistry under chronic IFNγ conditions with or without 48 h PDGF-BB treatment (Fig. 7a). Quantification of PDGFRβ staining had shown a reduction in protein expression upon treatment with PDGF-BB for 48 h, which is consistent with degradation of the receptor (Fig. 7b) and our previous observations (Fig. 2b). Chronic IFNγ treatment caused an even further reduction in PDGFRβ protein levels following PDGF-BB stimulation. There was also a significant reduction in αSMA expression in response to PDGF-BB as well as the previously observed effect of IFNγ treatment (Fig. 7c). Interestingly, PDGF-BB did not affect αSMA expression under chronic IFNγ conditions.

Closer examination of the cycling pattern of PDGFRβ by western blot showed a reduction in total PDGFRβ protein after 24 h of PDGF-BB treatment that was partially recovered after 48 h (Fig. 7d–f). However, recovery of PDGFRβ expression was not seen in the presence of IFNγ. Furthermore, analysis of αSMA expression demonstrated a PDGF-independent reduction with IFNγ treatment that was consistent across three cases and confirmed our prior observations. These data indicate that IFNγ may act to inhibit re-synthesis of PDGFRβ, and therefore, block downstream signalling required for proliferation and migration of pericytes after agonist-induced activation of the receptor. Again, the same paradigm was investigated in the presence of TNFα or IL-1β, and although there was a trend towards a decrease in PDGFRβ expression with either cytokine treatment, this result was not significantly different from vehicle treatment by either immunocytochemistry or western blot analysis (Additional file 7: Figure S7).

To determine if the negative regulation of PDGFRβ by IFNγ was acting at the translational or transcriptional level, we investigated mRNA expression after PDGF-BB treatment in the presence or absence of IFNγ. Although *PDGFRβ* expression remained relatively stable over time in the absence of the PDGF-BB, qRT-PCR revealed an increase in *PDGFRβ* gene expression at 48 and 72 h post-PDGF-BB treatment in the presence of IFNγ, although this was not statistically significant (Fig. 7g). *αSMA* mRNA expression closely followed results from protein analysis. *αSMA* expression was reduced by both PDGF-BB and IFNγ treatment at all time points (Fig. 7h). *Ki67* was increased in response to PDGF-BB at both 24 and 48 h after stimulation, and this effect was blocked by the presence of IFNγ (Fig. 7i). However, at 72 and 96 h after PDGF-BB treatment, no difference in proliferation is detected with IFNγ compared to vehicle. This is most likely due to either cells reaching confluency in the wells or saturation of the receptor signalling pathway.

## Discussion

Pericytes are a vital component of brain microvasculature and as such play an integral role in CNS homeostasis and BBB function [61]. There is now very convincing evidence that inflammation and an impaired BBB/BSCB can influence brain disease (reviewed [16, 62–64]). Previously, we and others have identified brain pericytes as active participants in the inflammatory response by upregulating gene and protein expression in response to inflammatory cues [22, 26, 51]. Here, for the first time, we have shown that chronic IFNγ modulates PDGFRβ pathway activation at both the proximal and distal end of signal transduction and thus impacts a crucial signalling pathway in human brain pericytes. Pericytes cultured in the presence of IFNγ for an extended period demonstrated increased membrane expression of PDGFRβ, as well as ligand-stimulated phosphorylation, and internalization. However, upon ligand-induced downregulation, PDGFRβ protein levels remained low, thereby reducing pericyte proliferation and migration. In this manner, IFNγ interferes with a key aspect of pericyte biology. The potential impacts of such an obstruction would be extremely detrimental to BBB function in health and disease. Previous in-depth studies have identified PDGF-BB and PDGFRβ as being necessary for pericyte coverage of the BBB in the developing CNS [27, 33]. Our data indicate that chronic IFNγ causes a transient increase in PDGFRβ membrane expression which therefore leads to an increase in ligand-induced receptor activation. This may be carried out through re-localization of the PDGFRβ to specialized areas of the cell (i.e., lipid rafts or non-rafts) that are differentially regulated for distinct cellular outputs [65]. The cellular purpose for this may be to maximize the responsiveness of pericytes in conditions that would require proliferation or migration, such as stroke [53, 66]. However, the presence of IFNγ, as demonstrated in our model, would negatively regulate this signal transduction.

Ultimately, elevated activation of the PDGFRβ by IFNγ resulted in a dampened PDGF-BB-dependent proliferation response. The effects of IFNγ on proliferation have been studied extensively and appear to be context and cell-type dependent (reviewed [67]). Data presented here identify a novel PDGF-BB-dependent mechanism for IFNγ-mediated inhibition of proliferation, as cell numbers and proliferation markers were unchanged by chronic IFNγ in the absence of PDGF-BB. Similarly, knockdown of PDGFRβ in our cultured human brain pericytes revealed a PDGF-BB-dependent block in proliferation without reducing the basal proliferative state and is supported by work in fibroblasts [68]. This is consistent with our theory that IFNγ blocks PDGFRβ re-synthesis, so that cells under chronic IFNγ conditions essentially become PDGFRβ-depleted, but only after initial ligand-induced degradation. Moreover, since transcript levels of *PDGFRβ* are not reduced by IFNγ treatment, this supports the conclusion that IFNγ modulates PDGFRβ expression specifically at the protein level.

We also observed an effect of IFNγ on pericyte migration in a scratch wound assay. The negative influence on cell migration was not specific to PDGF-BB-treated pericytes; therefore, we cannot conclude that this is PDGF-BB-dependent. In addition, since there was a block in proliferation by IFNγ, this may in itself appear to inhibit cell migration. However, previous studies of epithelial cell migration in response to IFNγ treatment had revealed modulation of cell focal adhesion proteins β1-integrin and vinculin at the leading edge of migrating cells [69]. This in turn resulted in a decreased rate of wound closure with IFNγ treatment in a scratch wound assay. Moreover, the differences in pericyte cell shape observed under IFNγ conditions support the idea that migration is also altered.

Our data suggest that chronic IFNγ modulates PDGFRβ turnover after ligand stimulation and therefore inhibits any subsequent receptor signalling. Indeed, degradation-defective mutants of the PDGFRβ demonstrate increased proliferation in response to ligand stimulation [60]. However, in addition to possible increases in receptor degradation, IFNγ may also cause inhibition of PDGFRβ protein translation, which was observed after PDGF-BB treatment. Compared to controls where PDGFRβ expression returns after 48 h, with IFNγ treatment, this does not occur. This phenomenon has not been previously investigated in regard to PDGFRβ expression; however, IFNγ has been recently shown to inhibit metabolism-related translation, in order to upregulate inflammatory-response proteins in macrophages [70]. In addition, TNFα and IL-1β were also shown to reduce PDGFRβ expression in fibroblasts and commercial pericytes; however, the mechanism behind this was not identified [37, 51]. We have seen that chronic treatment of primary human brain pericytes with either TNFα or IL-1β resulted in a decrease in PDGFRβ protein and mRNA expression (Additional file 1: Figure S1). This result was consistent with previous work in commercial human brain pericytes treated for 24 h with TNFα or IL-1β [51]. However, there was no change in PDGF-BB-stimulated PDGFRβ internalization (Additional file 4: Figure S4). And although PDGFRβ protein decreased over time with TNFα or IL-1β treatment, by both membrane and total PDGFRβ staining (Additional file 5: Figure S5A, B), PDGFRβ protein degradation was not significantly altered (Additional file 5: Figure S5C). Moreover, PDGF-BB-induced pericyte proliferation in the presence of TNFα or IL-1β was not different from vehicle conditions; therefore, we focused on the specific effects of IFNγ.

Nonetheless, the fact that individual cytokines can alter PDGFRβ expression via unique mechanisms will be important to consider when targeting this process in pathology. Chronic inflammation generally constitutes elevated concentrations of many cytokines, chemokines, and other pro-inflammatory modulators over an extended period of time (months to years). As such, studying the effects of a single cytokine alone has its limitations, however, is important for dissection of pathways and identification of molecular targets. It will be essential to also examine pericytes that have been exposed to a multitude of pro-inflammatory cues, such as those present in chronic inflammatory diseases. Now that we have established the response of human brain pericytes to both acute and chronic inflammatory cues, we can begin to investigate the effects of combinations of pro-inflammatory signals that are more representative of physiology in chronic disease conditions.

Initial observations of chronic IFNγ on pericyte markers revealed changes at the transcript level, curiously, with opposing effects on PDGFRβ and αSMA. Although PDGFRβ mRNA is constitutively expressed, growth factor deprivation has been shown to increase transcript levels [71]. Indeed, this is what we see in response to chronic IFNγ treatment, indicating that this is not dependent on stimulation with PDGF-BB.

The fact that IFNγ inhibits migration of pericytes both in the presence and absence of PDGF-BB also implies a PDGFRβ-independent mechanism. Our data also suggests that IFNγ-mediated inhibition of αSMA expression and migration is PDGF-independent and is in concordance with previous studies in smooth muscle cells and pericytes [72]. However, as there are a number of membrane scaffolding and filament proteins that make up the cytoskeletal machinery, IFNγ-mediated inhibition of migration by pericytes likely involves additional factors that have not been investigated here. In fact, modulation of cytoskeletal and focal adhesion protein arrangement has also been attributed to IFNγ-dependent migration inhibition in endothelial cells [69]. Interestingly, TNFα and IL-1β have also been shown to induce morphological changes in pericytes, namely a bipolar, linear morphology, which may also have an impact on how these cytokines can affect pericyte remodelling [73]. Therefore, alteration in cytoskeletal protein arrangement may indeed contribute to impaired healing and remodelling in response to injury leading to the leaky barrier properties seen in brain disease.

PDGFRβ expression in brain pericytes is required for BBB development and function [74]. However, this signalling pathway is also vital for the healing process in the cerebral vasculature. Pericyte-specific activation of PDGFRβ in response to either ischemia or traumatic brain injury has been shown in rodent models to be important in wound healing [53, 75]. Moreover, PDGFRβ deficiency resulted in increased infarct area, giving rise to the conclusion that its expression may also provide neuroprotection and contribute to more successful recovery [35, 66]. Interestingly, mice with PDGFRβ deficiency demonstrated vascular leakage with no difference in angiogenesis after ischemia, indicating that blood vessels can form but have limited barrier capacity [66]. An inability of pericytes to respond appropriately to PDGF-BB released by endothelial cells after stress or injury may have severe consequences. Although ischemia and traumatic brain injury are extreme examples, many factors can contribute to more subtle BBB damage, such as metabolic diseases (diabetes and obesity), genetics (*NOTCH3*), and vascular risk factors [76–78]. These small infractions accumulated over time in the presence of a pro-inflammatory component would negatively regulate the expression of PDGFRβ.

Inflammation has long been considered a contributor to BBB breakdown, though the reasons for this have not been clear. Now, we have evidence that not only pericytes play an important role in transmitting the inflammatory response by the expression of cytokines, chemokines, and cell surface adhesion molecules but also the sustainability of basic pericyte functions is vulnerable to chronic inflammatory stress. In the case of IFNγ, attenuation of PDGF-BB signalling through decreased PDGFRβ expression may result in a weakened response of pericytes; therefore, damage to the BBB/BSCB would be left unrepaired. Investigations of attenuated PDGFRβ and the effects on pericyte survival and function in a whole animal context should be examined with an inducible knockout of PDGFRβ in brain pericytes. However, studies using cultured human brain pericytes can offer understanding of specific mechanistic properties of pericyte function and help to identify molecular targets for improving pericyte survival and proliferation under chronic inflammatory conditions. With these factors considered, our in vitro results regarding PDGFRβ pathway activation and protein expression may have profound implications for understanding pericyte dysfunction. Knowledge of the exact mechanisms of this and how it may be modulated pharmacologically would be beneficial for treatment of disease where BBB impairment is present.

## Conclusions

PDGFRβ is critical for pericyte function but is reduced in brain disorders that exhibit BBB and BSCB damage. We have identified a mechanism by which chronic IFNγ reduces pericyte signalling through the PDGFRβ pathway and potentially BBB impairment in vascular-related brain dysfunction.

## Additional files

Additional file 1: Figure S1. Pericytes were treated for four consecutive days (once every 24 h) with either vehicle (Veh), TNFα (5 ng/mL), or IL-1β (1 ng/mL) as depicted in Fig. 1a. Cells were then fixed and total cells counted from Hoechst labelled nuclei (A). The integrated intensity of the staining PDGFRβ or αSMA staining was normalized to cell number (Hoechst) and vehicle conditions (B,C), quantified from triplicate wells, and plotted as mean ± s.e.m (*n* = 3), ***(*p* < 0.001), *(*p* < 0.05) (Student’s *t* test). (D, E) mRNA from pericytes treated as in (A) was analysed by qRT-PCR. Inflammatory target genes (*IP-10*, *MCP-1*, *COX2*, *ICAM-1*, *CD74*) (D) and pericyte marker and proliferation marker genes (*PDGFRβ*, *αSMA*, and *Ki67*) (E) expression were normalized to *GAPDH* and plotted as a fold change from vehicle (set to 1) (2^ΔΔCT) ± s.e.m (*n* = 3), ****(*p* < 0.0001), ***(*p* < 0.001), **(*p* < 0.01), *(*p* < 0.05) by a Mann-Whitney, non-parametric test of ΔCT values. Note: Control data are from the same experiments as Fig. 1. (TIF 1383 kb) Additional file 2: Figure S2. Repeats of additional cases from Fig. 2: (A) Pericytes were serum starved for 2 h and then treated with vehicle (lanes 1 and 7) or PDGF-BB (100 ng/mL) (lanes 2–6, and 8) for the indicated times and analysed by SDS-PAGE as in Fig. 2. Representative blots from two additional cases are shown. (B–F) Blots from (A) were analysed and quantified with Image Studio™. Phosphorylated PDGFRβ (Tyr751) (p-PDGFRβ) (B), PDGFRβ (C), and PDGF-BB (F) were normalized to GAPDH; phosphorylated Akt (Ser473) (p-Akt) (D) and phosphorylated ERK (Tyr204) (p-ERK) (E) were normalized to total Akt and ERK, respectively. (TIF 15997 kb) Additional file 3: Figure S3. Repeats of additional cases from Fig. 3(b–e): Pericytes were treated for four consecutive days (once every 24 h) with either vehicle (Veh) or IFNγ (1 ng/mL). After 96 h total treatment, cells were serum starved for 2 h and then treated with vehicle (−) or PDGF-BB (100 ng/mL) for 30 min as in Fig. 3. (a, c) Representative western blots of treated pericyte from two additional cases. (B, D) Bands were quantified with Image Studio™ and normalized to vehicle control. p-PDGFRβ was normalized to total PDGFRβ, and p-Akt and p-ERK were normalized to total Akt and ERK, respectively. (TIF 2398 kb) Additional file 4: Figure S4. Pericytes were treated for four consecutive days (once every 24 h) with either vehicle (Veh), TNFα (5 ng/mL), or IL-1β (1 ng/mL). After 96 h total treatment, cells were serum starved for 2 h and then treated with vehicle (−) or PDGF-BB (100 ng/mL) for 30 min. PDGFRβ puncta were quantified using MetaXpress™ software and normalized to cell number and vehicle control and plotted as mean ± s.e.m. (*n* = 3), ***(*p* < 0.001), *(*p* < 0.05) (two-way ANOVA). Note: Control data are from the same experiments as Fig. 3g. (TIF 723 kb) Additional file 5: Figure S5. (A, B) Pericytes were treated for 24, 48, 72, or 96 h (more cytokines added once every 24 h to appropriate wells) with vehicle (Veh), TNFα (5 ng/mL), or IL-1β (1 ng/mL). Quantification of total PDGFRβ (white bars) and membrane PDGFRβ (black bars) staining intensity per cell from TNFα treated (A) or IL-1β treated (B) was normalized to 0 h time point, plotted as mean ± s.e.m. (*n* = 2), *(*p* < 0.05) (ANOVA). (TIF 1027 kb) Additional file 6: Figure S6. Pericytes were treated for four consecutive days (once every 24 h) with either vehicle (Veh), TNFα (5 ng/mL), or IL-1β (1 ng/mL). After 48 h of cytokine treatment, cells were treated with either vehicle or PDGF-BB (10 ng/mL) to measure the PDGF-BB-induced proliferative response (A, B). This was done in two ways: after 96 h total treatment, cells were fixed, labelled with a Ki67 antibody and Hoechst (A, C); alternatively, EdU was added to measure cell proliferation over the final 24 h of the experiment (B, C). Positive cells of the total cells measured by Hoechst were quantified and plotted as mean ± s.e.m. (*n* = 3), ****(*p* < 0.0001), ***(*p* < 0.001), *(*p* < 0.05) from a two-way ANOVA. (TIF 1034 kb) Additional file 7: Figure S7. (A, B) Pericytes were treated for four consecutive days (once every 24 h) with either vehicle (Veh), TNFα (5 ng/mL), or IL-1β (1 ng/mL). After 48 h of cytokine treatment, cells were treated with either vehicle or PDGF-BB (10 ng/mL) to measure PDGFRβ and αSMA expression by immunocytochemistry. Quantification of PDGFRβ (A) and αSMA (B) staining, mean ± s.e.m. (*n* = 3), ****(*p* < 0.0001), ***(*p* < 0.001), *(*p* < 0.05) (two-way ANOVA). (C) Pericytes were treated for three or four consecutive days (once every 24 h) with either vehicle (Veh), TNFα (5 ng/mL), or IL-1β (1 ng/mL). After 48 h, cells were treated with PDGF-BB (10 ng/mL) for either 24 or 48 h. Western blot band intensity of PDGFRβ, αSMA, and GAPDH were quantified, normalized to GAPDH, and plotted as mean ± s.e.m. (*n* = 3), and differences were not significant (two-way ANOVA). (TIF 1163 kb)

## Abbreviations

αSMA: Alpha smooth muscle actin
AD: Alzheimer’s disease
BBB: Blood-brain barrier
CNS: Central nervous system
CD74: Cluster of differentiation 74
COX2: Cyclooxygenase 2
ERK: Extracellular signal-regulated kinase
GAPDH: Glyceraldehyde-3-phosphate dehydrogenase
IFNγ: Interferon gamma
IL-1β: Interleukin-1beta
ICAM-1: Intracellular adhesion molecule
IP-10: Interferon-inducible protein-10
MAPK: Mitogen-activated protein kinase
MCP-1: Monocyte chemotactic protein-1
MS: Multiple sclerosis
PD: Parkinson’s disease
PI3K: Phosphotidylinositol-3-kinase
PDGFRβ: Platelet-derived growth factor receptor-beta
TNFα: Tumour necrosis factor alpha
