# Supplementary figures and images for: Interferon-γ blocks signalling through PDGFRβ in human brain pericytes
Source: J Neuroinflammation. 2016 Sep 21;13:249. doi: 10.1186/s12974-016-0722-4 (PMC5031293; doi:10.1186/s12974-016-0722-4)

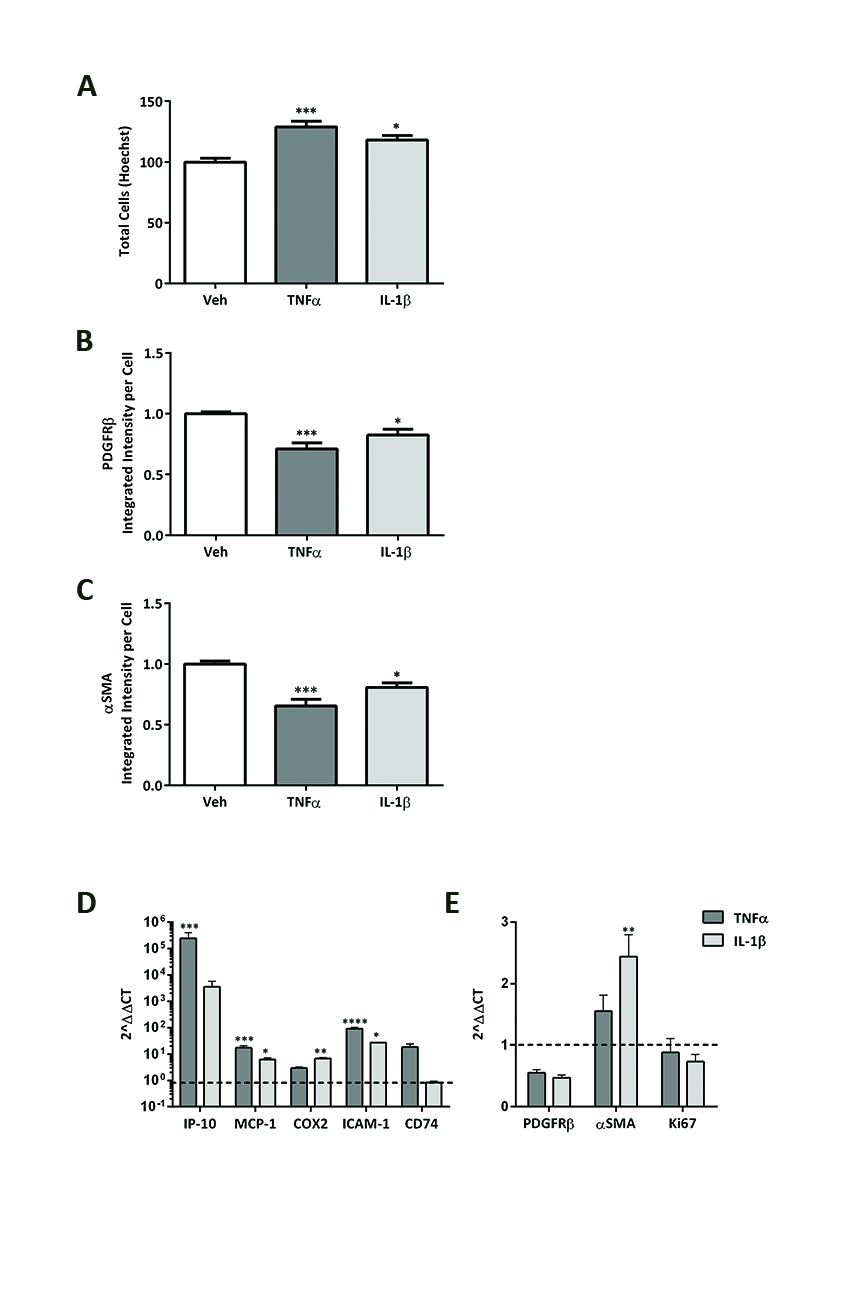

Supplement: Additional file 1: Figure S1. — Pericytes were treated for four consecutive days (once every 24 h) with either vehicle (Veh), TNFα (5 ng/mL), or IL-1β (1 ng/mL) as depicted in Fig. 1a. Cells were then fixed and total cells counted from Hoechst labelled nuclei (A). The integrated intensity of the staining PDGFRβ or αSMA staining was normalized to cell number (Hoechst) and vehicle conditions (B,C), quantified from triplicate wells, and plotted as mean ± s.e.m (n = 3), ***(p < 0.001), *(p < 0.05) (Student’s t test). (D, E) mRNA from pericytes treated as in (A) was analysed by qRT-PCR. Inflammatory target genes (IP-10, MCP-1, COX2, ICAM-1, CD74) (D) and pericyte marker and proliferation marker genes (PDGFRβ, αSMA, and Ki67) (E) expression were normalized to GAPDH and plotted as a fold change from vehicle (set to 1) (2^ΔΔCT) ± s.e.m (n = 3), ****(p < 0.0001), ***(p < 0.001), **(p < 0.01), *(p < 0.05) by a Mann-Whitney, non-parametric test of ΔCT values. Note: Control data are from the same experiments as Fig. 1. (TIF 1383 kb) [file 12974_2016_722_MOESM1_ESM.tif]

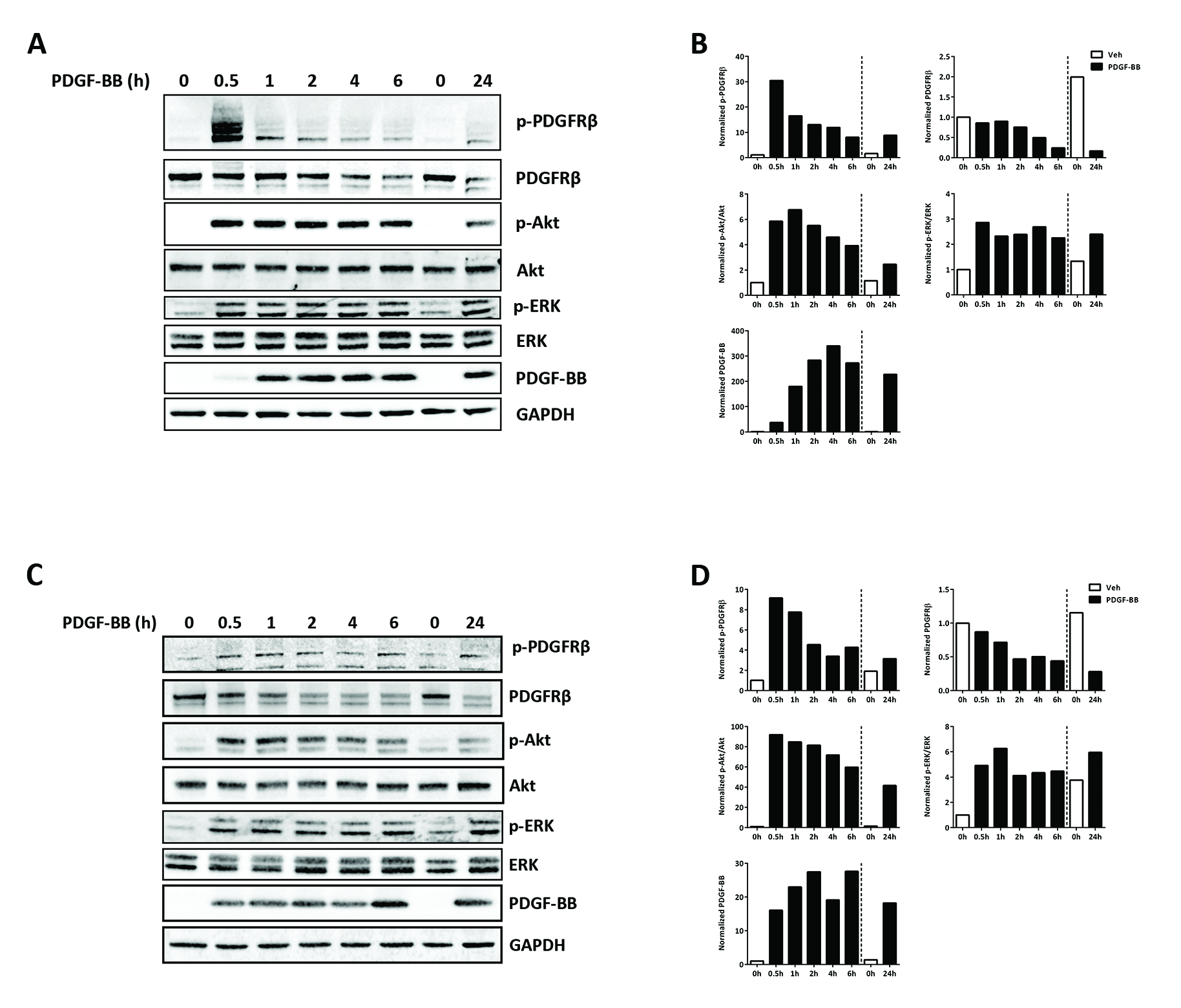

Supplement: Additional file 2: Figure S2. — Repeats of additional cases from Fig. 2: (A) Pericytes were serum starved for 2 h and then treated with vehicle (lanes 1 and 7) or PDGF-BB (100 ng/mL) (lanes 2–6, and 8) for the indicated times and analysed by SDS-PAGE as in Fig. 2. Representative blots from two additional cases are shown. (B–F) Blots from (A) were analysed and quantified with Image Studio™. Phosphorylated PDGFRβ (Tyr751) (p-PDGFRβ) (B), PDGFRβ (C), and PDGF-BB (F) were normalized to GAPDH; phosphorylated Akt (Ser473) (p-Akt) (D) and phosphorylated ERK (Tyr204) (p-ERK) (E) were normalized to total Akt and ERK, respectively. (TIF 15997 kb) [file 12974_2016_722_MOESM2_ESM.tif]

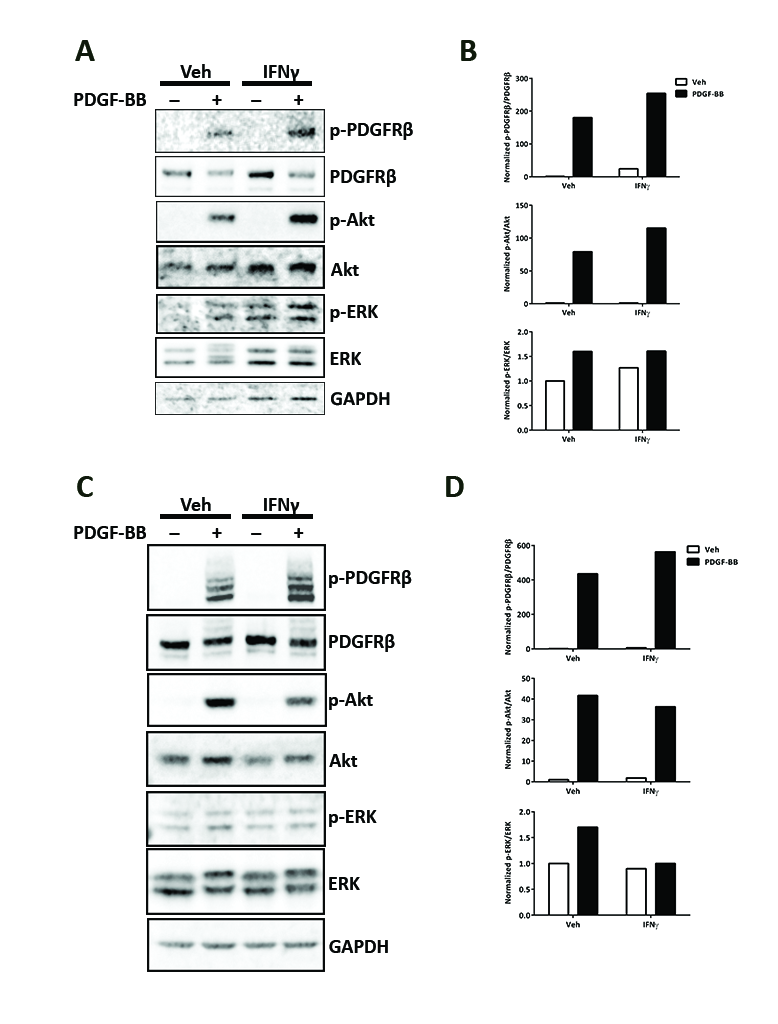

Supplement: Additional file 3: Figure S3. — Repeats of additional cases from Fig. 3(b–e): Pericytes were treated for four consecutive days (once every 24 h) with either vehicle (Veh) or IFNγ (1 ng/mL). After 96 h total treatment, cells were serum starved for 2 h and then treated with vehicle (−) or PDGF-BB (100 ng/mL) for 30 min as in Fig. 3. (a, c) Representative western blots of treated pericyte from two additional cases. (B, D) Bands were quantified with Image Studio™ and normalized to vehicle control. p-PDGFRβ was normalized to total PDGFRβ, and p-Akt and p-ERK were normalized to total Akt and ERK, respectively. (TIF 2398 kb) [file 12974_2016_722_MOESM3_ESM.tif]

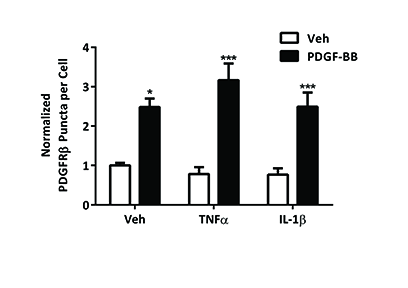

Supplement: Additional file 4: Figure S4. — Pericytes were treated for four consecutive days (once every 24 h) with either vehicle (Veh), TNFα (5 ng/mL), or IL-1β (1 ng/mL). After 96 h total treatment, cells were serum starved for 2 h and then treated with vehicle (−) or PDGF-BB (100 ng/mL) for 30 min. PDGFRβ puncta were quantified using MetaXpress™ software and normalized to cell number and vehicle control and plotted as mean ± s.e.m. (n = 3), ***(p < 0.001), *(p < 0.05) (two-way ANOVA). Note: Control data are from the same experiments as Fig. 3g. (TIF 723 kb) [file 12974_2016_722_MOESM4_ESM.tif]

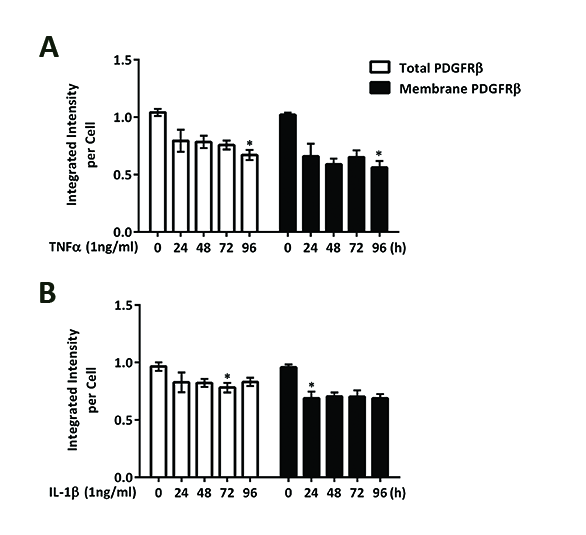

Supplement: Additional file 5: Figure S5. — (A, B) Pericytes were treated for 24, 48, 72, or 96 h (more cytokines added once every 24 h to appropriate wells) with vehicle (Veh), TNFα (5 ng/mL), or IL-1β (1 ng/mL). Quantification of total PDGFRβ (white bars) and membrane PDGFRβ (black bars) staining intensity per cell from TNFα treated (A) or IL-1β treated (B) was normalized to 0 h time point, plotted as mean ± s.e.m. (n = 2), *(p < 0.05) (ANOVA). (TIF 1027 kb) [file 12974_2016_722_MOESM5_ESM.tif]

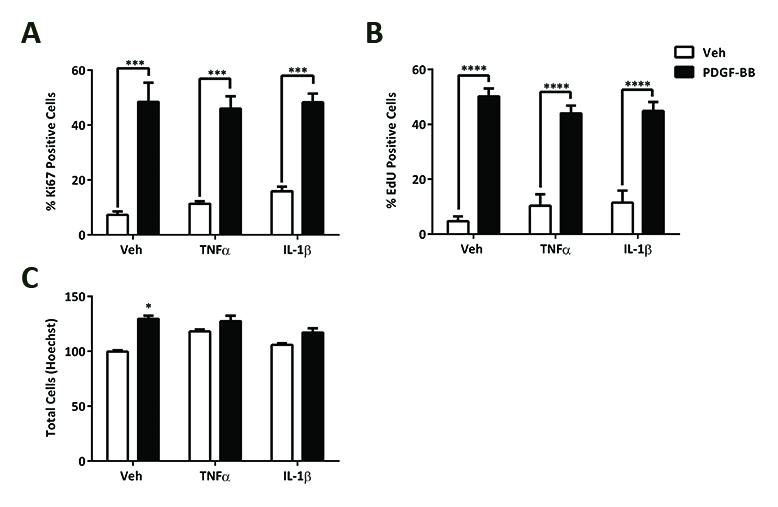

Supplement: Additional file 6: Figure S6. — Pericytes were treated for four consecutive days (once every 24 h) with either vehicle (Veh), TNFα (5 ng/mL), or IL-1β (1 ng/mL). After 48 h of cytokine treatment, cells were treated with either vehicle or PDGF-BB (10 ng/mL) to measure the PDGF-BB-induced proliferative response (A, B). This was done in two ways: after 96 h total treatment, cells were fixed, labelled with a Ki67 antibody and Hoechst (A, C); alternatively, EdU was added to measure cell proliferation over the final 24 h of the experiment (B, C). Positive cells of the total cells measured by Hoechst were quantified and plotted as mean ± s.e.m. (n = 3), ****(p < 0.0001), ***(p < 0.001), *(p < 0.05) from a two-way ANOVA. (TIF 1034 kb) [file 12974_2016_722_MOESM6_ESM.tif]

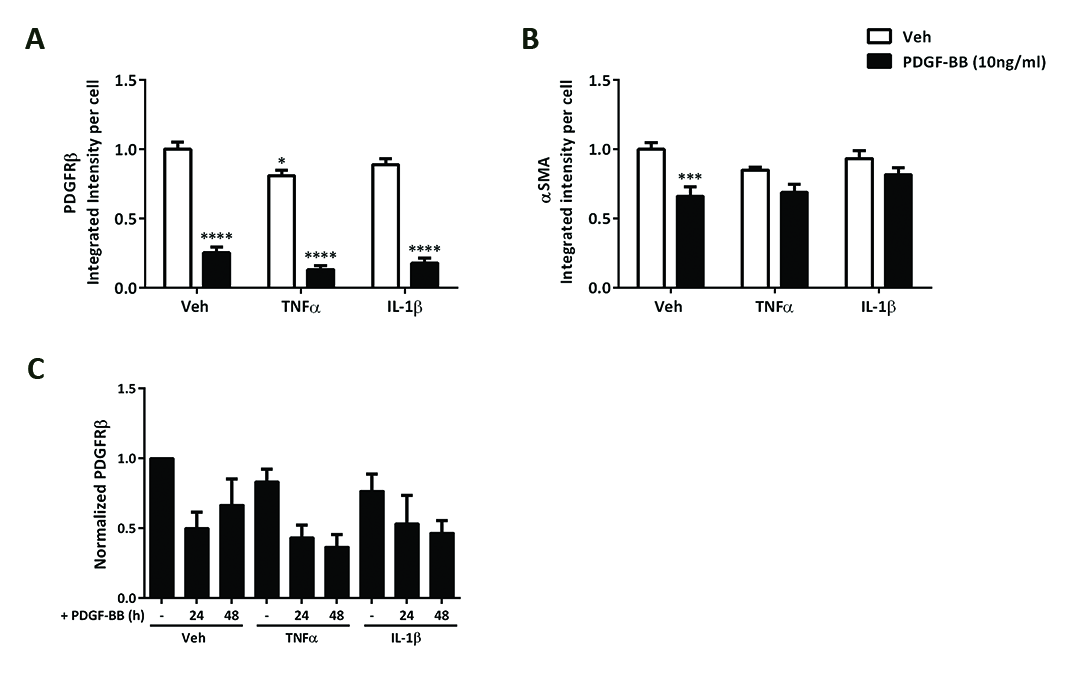

Supplement: Additional file 7: Figure S7. — (A, B) Pericytes were treated for four consecutive days (once every 24 h) with either vehicle (Veh), TNFα (5 ng/mL), or IL-1β (1 ng/mL). After 48 h of cytokine treatment, cells were treated with either vehicle or PDGF-BB (10 ng/mL) to measure PDGFRβ and αSMA expression by immunocytochemistry. Quantification of PDGFRβ (A) and αSMA (B) staining, mean ± s.e.m. (n = 3), ****(p < 0.0001), ***(p < 0.001), *(p < 0.05) (two-way ANOVA). (C) Pericytes were treated for three or four consecutive days (once every 24 h) with either vehicle (Veh), TNFα (5 ng/mL), or IL-1β (1 ng/mL). After 48 h, cells were treated with PDGF-BB (10 ng/mL) for either 24 or 48 h. Western blot band intensity of PDGFRβ, αSMA, and GAPDH were quantified, normalized to GAPDH, and plotted as mean ± s.e.m. (n = 3), and differences were not significant (two-way ANOVA). (TIF 1163 kb) [file 12974_2016_722_MOESM7_ESM.tif]
